# Supplementary material for: Myoepithelial progenitors as founder cells of hyperplastic human breast lesions upon PIK3CA transformation
Source: Commun Biol. 2022 Mar 10;5:219. doi: 10.1038/s42003-022-03161-x (PMC8913783; doi:10.1038/s42003-022-03161-x)
Supplement: Supplementary file 2 — Supplementary Information [file 42003_2022_3161_MOESM2_ESM.pdf]

Supplementary Information

Supplementary Fig. 1. Spatial mapping of MEP progenitors at the single cell level.

a) Heatmap displays relative gene expression levels of top 10 DEGs per cluster in 18,678 MEP cells grouped by cluster, color-coded according to t-SNE plot in Fig. 1a (higher, yellow; lower, pink). b) Bar graphs show significant overlap (-log(p-value)) between DEGs of cluster 8 (top) and cluster 9 (bottom) with genes sets related to stroma and vasculature/immune cells, respectively, from the Molecular Signatures Database. c) Cluster-assigned bubble plot of luminal (*KRT15*, *KRT19*, *SLPI*, *LTF*, *CD24*) and MEP markers (*KRT5*, *KRT14*, *KRT17*, *MYLK*, *ACTA2*, *TP63*) shows up-regulation of luminal genes in cluster 7 and MEP genes in clusters 0 to 6 indicating that the first seven clusters are true MEP clusters.

Supplementary Fig. 1. Spatial mapping of MEP progenitors at the single cell level

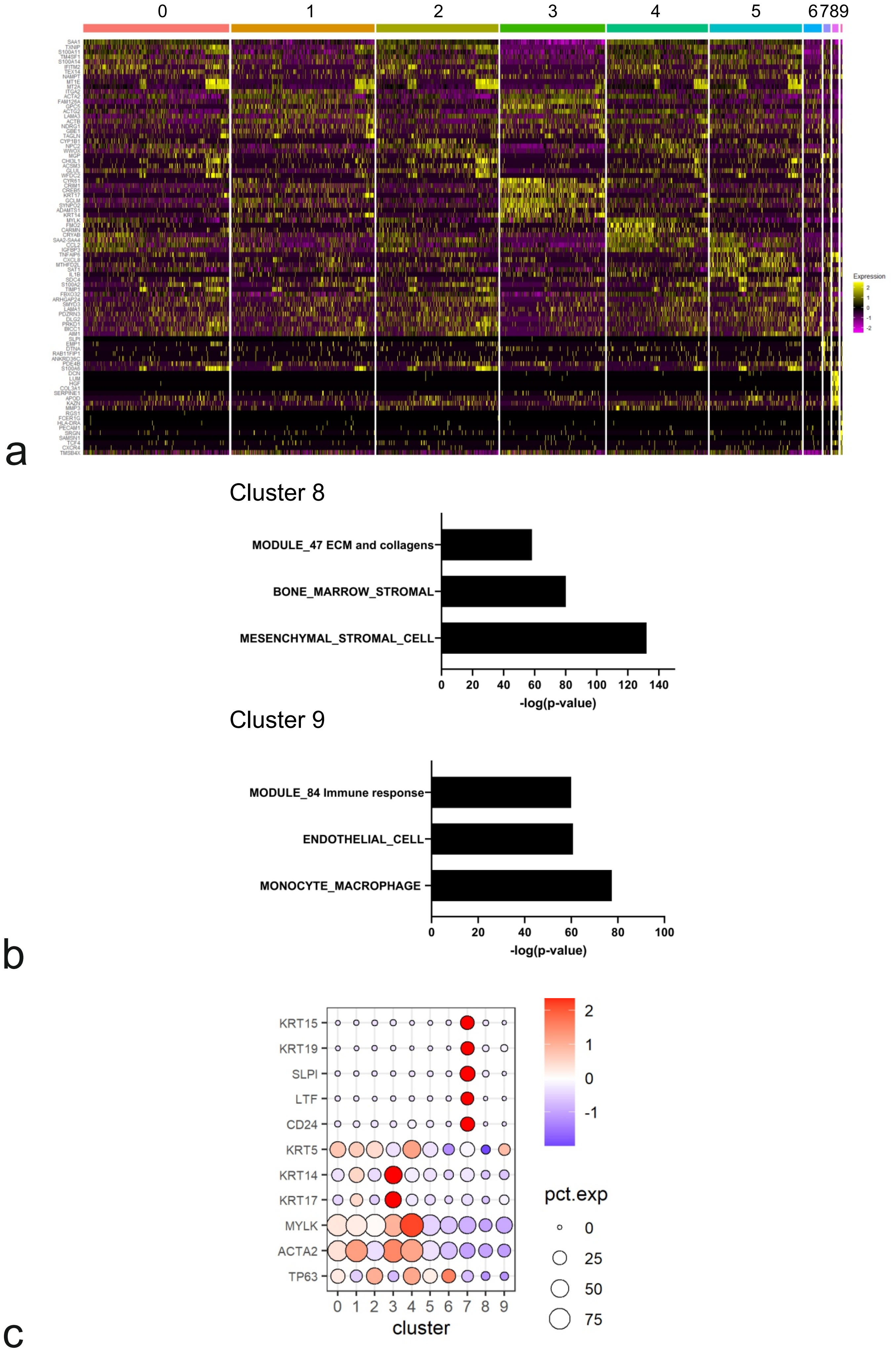

Supplementary Fig. 2. CD200 is a marker for a distinct MEP subpopulation.

a) Representative contour FACS plots of pre-sorted myoepithelial cells from eight different human normal breast biopsies illustrate biopsy-dependent variability in CD271 and CD200 expression. b) (upper panel) Multicolor images of cryosections of TDLU, duct (20x), and terminal duct (TD, 40x) stained for SMA with HHF-35 (green), CD200 (red) and nuclei (blue) show accumulation of CD200^{high} cells in terminal duct versus alveoli and duct (scale bar, 100 μm for TDLU and duct and 50 μm for terminal duct). (lower panel) Schematic overview of distribution of CD200^{low} (blue) and CD200^{high} (red) MEP in alveoli (Alv) and terminal duct (TD) of micro-collected TDLU and duct.

Supplementary Fig. 2. CD200 is a marker for a distinct MEP subpopulation

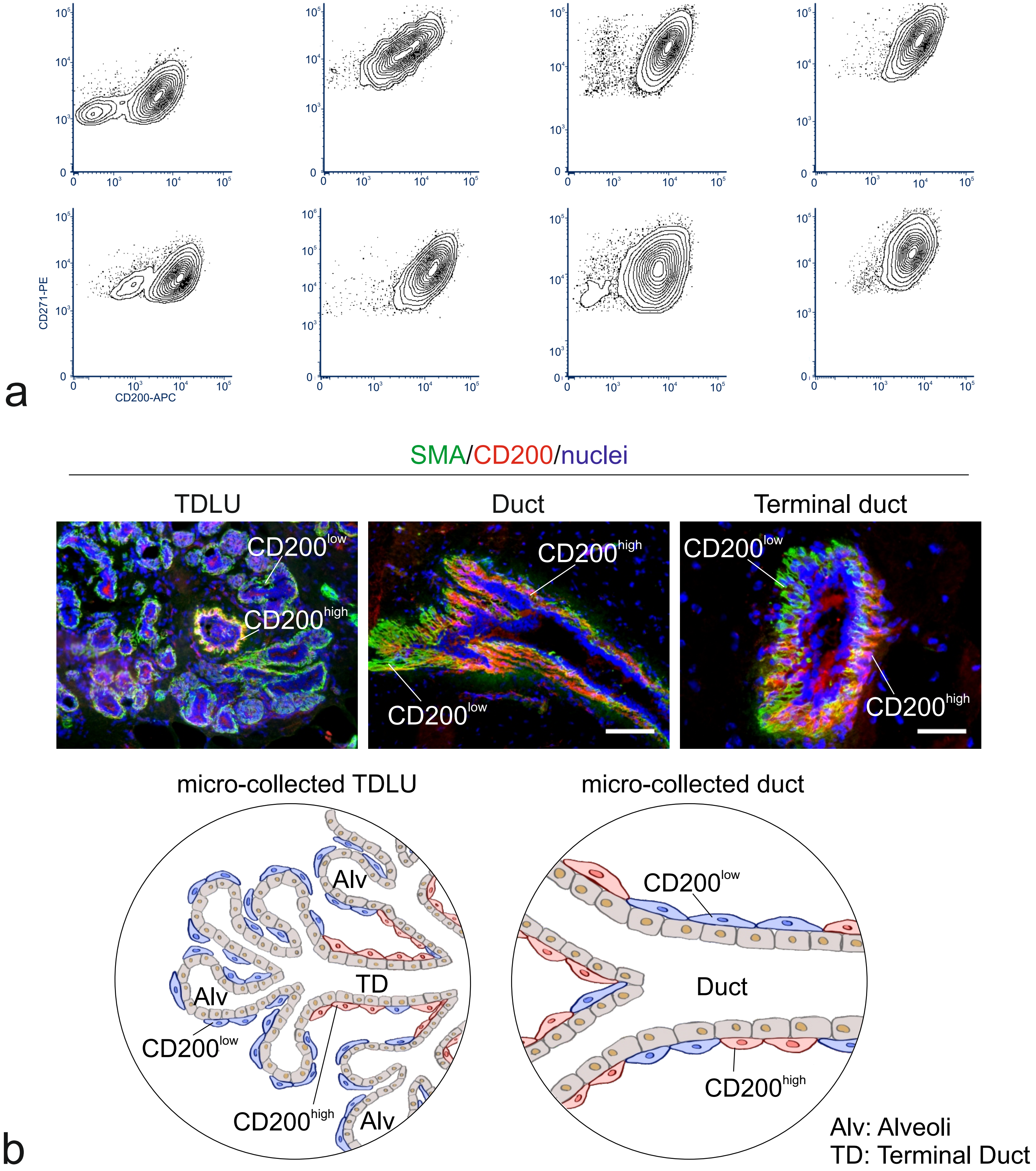

Supplementary Fig. 3. Myo$^+$ conditions support ground state of MEP progenitors poised for luminal differentiation.

a) Bar graph depicts quantification of frequency of α-SMA-positive cells (% α-SMA positive cells) in passage 1 to 4 of Trop2$^+$/CD271^{high} myoepithelial cells from a biopsy with initial high frequency of α-SMA MEP on iHBFC^{CD105} feeders. Under control conditions (Myo, black) α-SMA expression decreases in passages 3 and 4, while in Myo medium with IFNα and 1% oxygen (medium grey), a significantly higher expression of α-SMA is seen in passage 3 and 4 compared to Myo medium (*$p<0.05$ for P3 and ***$p<0.005$ for P4 by two-way ANOVA and Tukey's multiple comparison test). When cultured with IFNα, RepSox, and under hypoxic conditions (Myo$^+$, dark grey), α-SMA expression is significantly increased in all passages compared to Myo medium (***$p<0.005$ by two-way ANOVA and Tukey's multiple comparison test). Bars indicate mean ± standard deviation (n = 3 regions in MEP cell culture). b) Micrographs of primary CD200^{high} MEP cells stained for K14 (green), K19 (red) and α-SMA (red) show maintenance of K14$^+$/K19$^-$ and K14$^+$/α-SMA$^+$ MEP progenitors in Myo$^+$ conditions (Myo$^+$ (Ground state)). Upon induced luminal differentiation in MEGM with A83-01 (MEGM$^+$ (Differentiated)), the K14$^+$/K19$^-$ phenotype is maintained (first column), but in addition, colonies comprising K14$^+$/K19$^+$ (second column), but not K14$^-$/K19$^+$, appear (scale bar, 100 μm).

Supplementary Fig. 3. Myo⁺ conditions support ground state of MEP progenitors poised for luminal differentiation

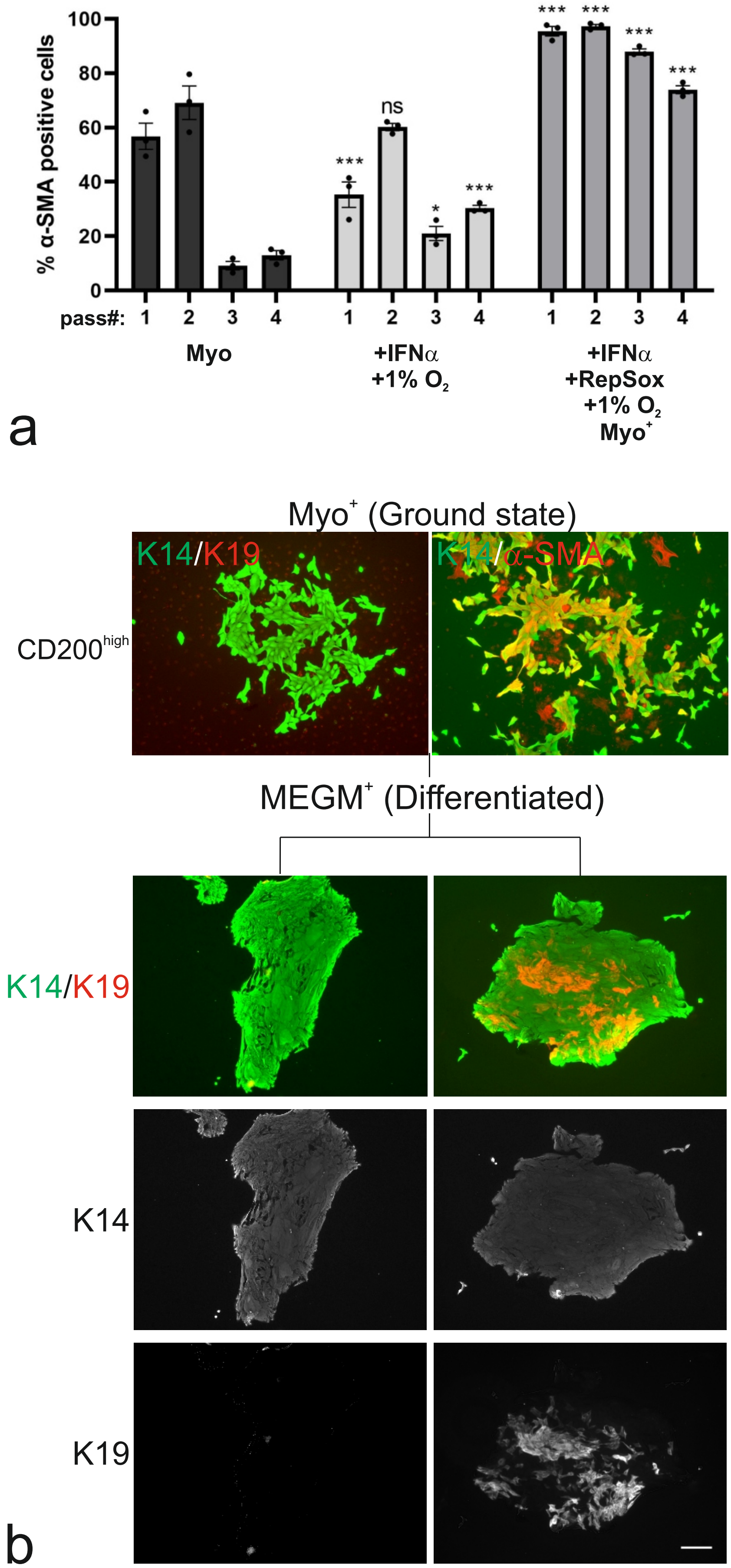

Supplementary Fig. 4. CD200^{low} and CD200^{high} MEP progenitors have distinct differentiation repertoires.

a) Bar graphs of quantification of frequency (% cells) of myodifferentiated (α-SMA, light colors), $K14^+/K19^+$ (medium) and $K14^-/K19^+$ (dark colors) cells among CD200^{low} (blue) and CD200^{high} (red) cells in ground state (Myo$^+$) and upon differentiation (MEGM$^+$), respectively, show a significantly higher frequency of luminal $K14^-/K19^+$ cells in CD200^{low} cultures. Bars indicate mean ± SEM ($p<0.01$ by two-tailed Mann-Whitney test, n = 7 for Myo$^+$ and n = 6 for MEGM$^+$ experiments representing four biopsies). b) Bar graph of fold change of normalized gene expression by RT-qPCR. Upon differentiation (MEGM$^+$, dark shade) CD200^{low} (blue) and CD200^{high} (red) both respond with reduced *KRT14* and *TP63* and increased *EPCAM* expression, but a significantly higher upregulation of *ELF5* and *KRT19* is observed in CD200^{low}. Bars indicate mean + SEM (***$p<0.005$ by two-way ANOVA and Tukey's multiple comparison test, n = 3). c) Micrograph of primary CD200^{low} MEP cells stained for K19 (green) and ER (red) upon culture in ER-supporting conditions shows that CD200^{low} MEP cells are able to express ER (scale bar, 100 μm).

Supplementary Fig. 4. CD200^low and CD200^high MEP progenitors have distinct differentiation repertoires

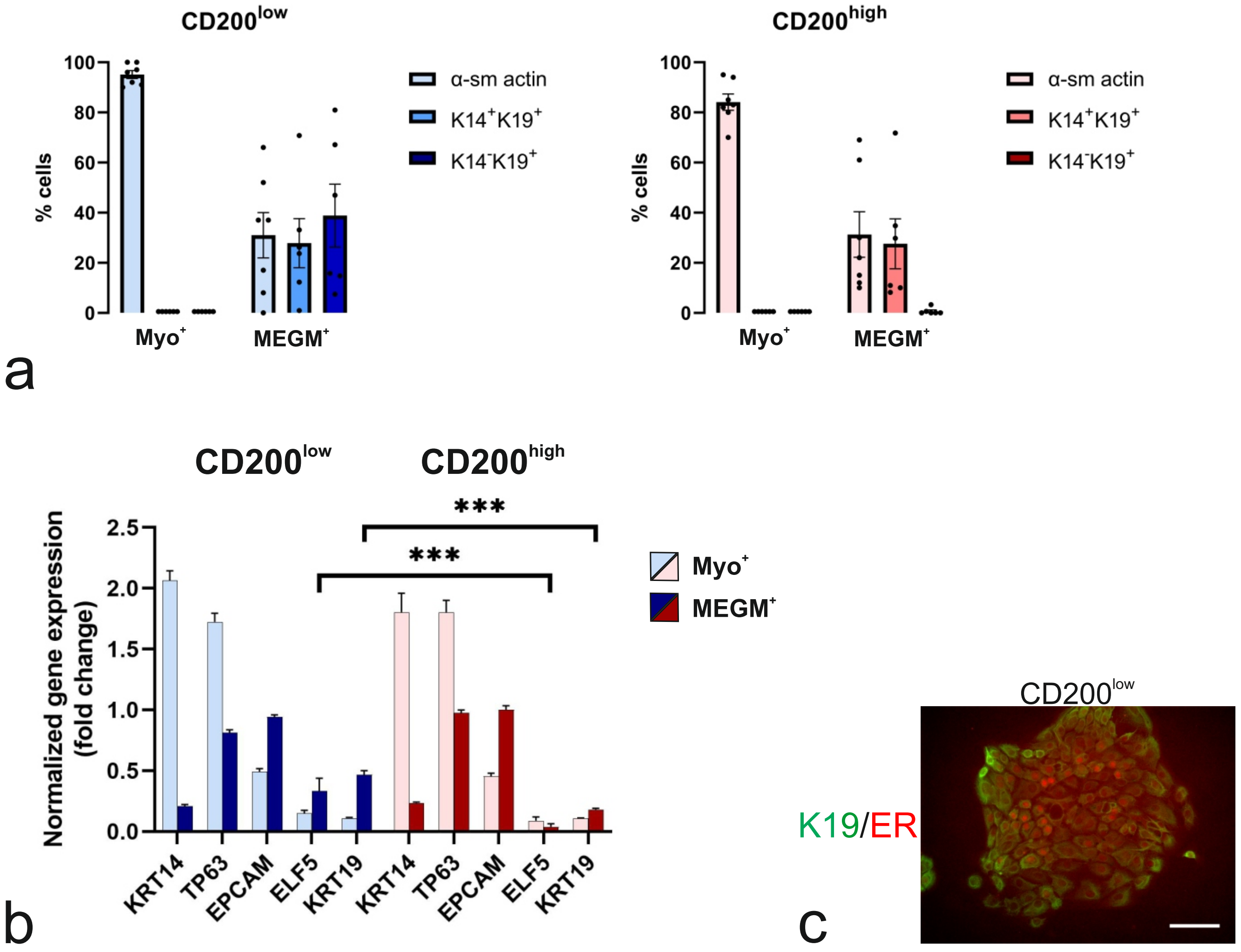

***Supplementary Fig. 5. A sequential transduction protocol provides identical growth rates with and without mutant* PIK3CA.**

a) Line drawing of passage number (P) for transduction of CD200^{low} (blue) and CD200^{high} (red) with *hTERT*, shp53 and *PIK3CA*^{H1047R}, respectively, with indication of the number of passages the cell strains have been cultured. b) Diagram of the calculated population doublings as a function of time (days) for untransfected CD200^{low} (blue, diamond) and CD200^{high} (red, diamond), in comparison with *hTERT* transfected (squares), *hTERT*-shp53 transfected (triangles) and *hTERT*-shp53-*PIK3CA*^{H1047R} (circles). *hTERT* extends the lifespan of the cells, albeit not increasing the growth rate, while further transfection with either shp53, or shp53 and *PIK3CA*^{H1047R} decreases the population doubling time considerably and to a similar degree in both CD200^{low} and CD200^{high}. c) Bar graphs of fold change of normalized gene expression in CD200^{low} and CD200^{high} strains by qRT-PCR confirm significant silencing of *TP53* in *hTERT*-shp53 and *hTERT*-shp53-*PIK3CA*^{H1047R} transfected cell strains as compared to *hTERT* transfected cell strains, and a significantly higher expression of *PIK3CA* in hTERT-shp53-*PIK3CA*^{H1047R} as compared to *hTERT* and *hTERT*-shp53 transfected cells strains. Bars indicate mean ± SEM (***p<0.005 by two-way ANOVA and Tukey´s multiple comparison test, n = 3).

***Supplementary Fig. 5. A sequential transduction protocol provides identical growth rates with and without mutant* PIK3CA**

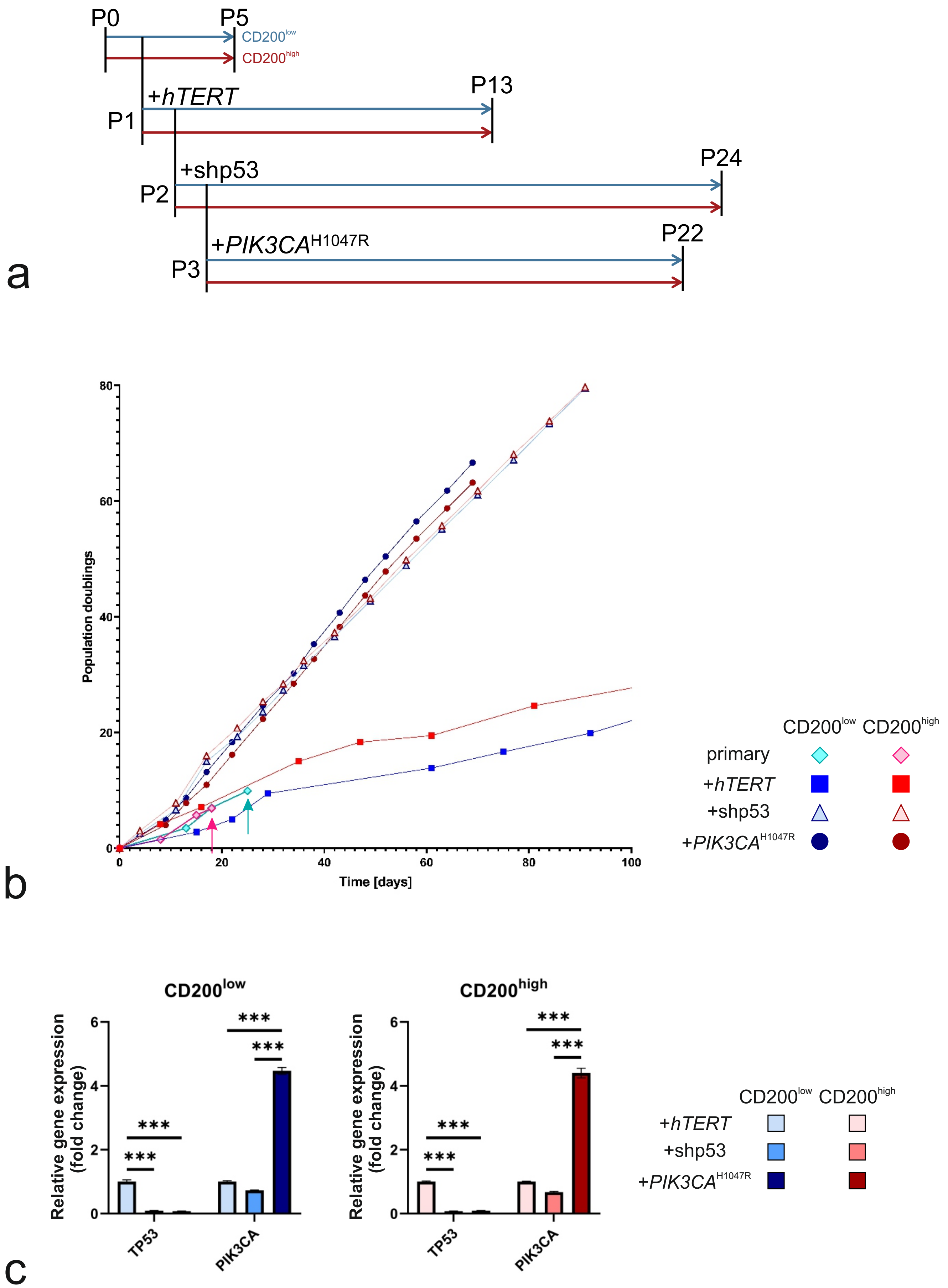

Supplementary Fig. 6. Mutant* PIK3CA *destabilizes ground state phenotype without affecting differentiation repertoire.

a) Micrographs of fluorescence stainings of (left) CD200^{low}-*hTERT*-shp53 and (right) CD200^{high}-*hTERT*-shp53 without (-) or with (+) mutant *PIK3CA* (*PIK3CA*^{H1047R}) in Myo^{+} (Ground state) show maintenance of K14 (green), p63 (red), and K17 (red) expression upon *PIK3CA*^{H1047R} expression in both cell lines (scale bar, 50 μm). b) Bar graph of fold change of normalized relative gene expression in CD200^{low}-*hTERT*-shp53-*PIK3CA*^{H1047R} (blue) and CD200^{high}-*hTERT*-shp53-*PIK3CA*^{H1047R} (red) lines assessed by RT-qPCR shows higher expression of cluster 3 markers *CD200*, *AMIGO2*, *ACTA2*, and *FAM126A* is maintained in the CD200^{high} cell line compared to the CD200^{low} cell line. Similarly, CD200^{low}-*hTERT*-shp53-*PIK3CA*^{H1047R} retains higher expression of *TXNIP*, *RARRES1*, and *TNFSF10* compared to CD200^{high}-*hTERT*-shp53-*PIK3CA*^{H1047R}. Bars indicate mean ± SEM (*** $p<0.005$ by multiple unpaired t tests with Bonferroni correction, n = 4 cultures in different passages). c) Representative micrographs of peroxidase staining of (left) CD200^{low}-*hTERT*-shp53-*PIK3CA*^{H1047R} and (right) CD200^{high}-*hTERT*-shp53-*PIK3CA*^{H1047R} after differentiation show a relatively broad expression of the luminal marker FOXA1, while expression of the ER-associated protein GATA3 is restricted to CD200^{low}-*hTERT*-shp53-*PIK3CA*^{H1047R} (scale bar, 50 μm).

Supplementary Fig. 6. Mutant* PIK3CA *destabilizes ground state phenotype without affecting differentiation repertoire

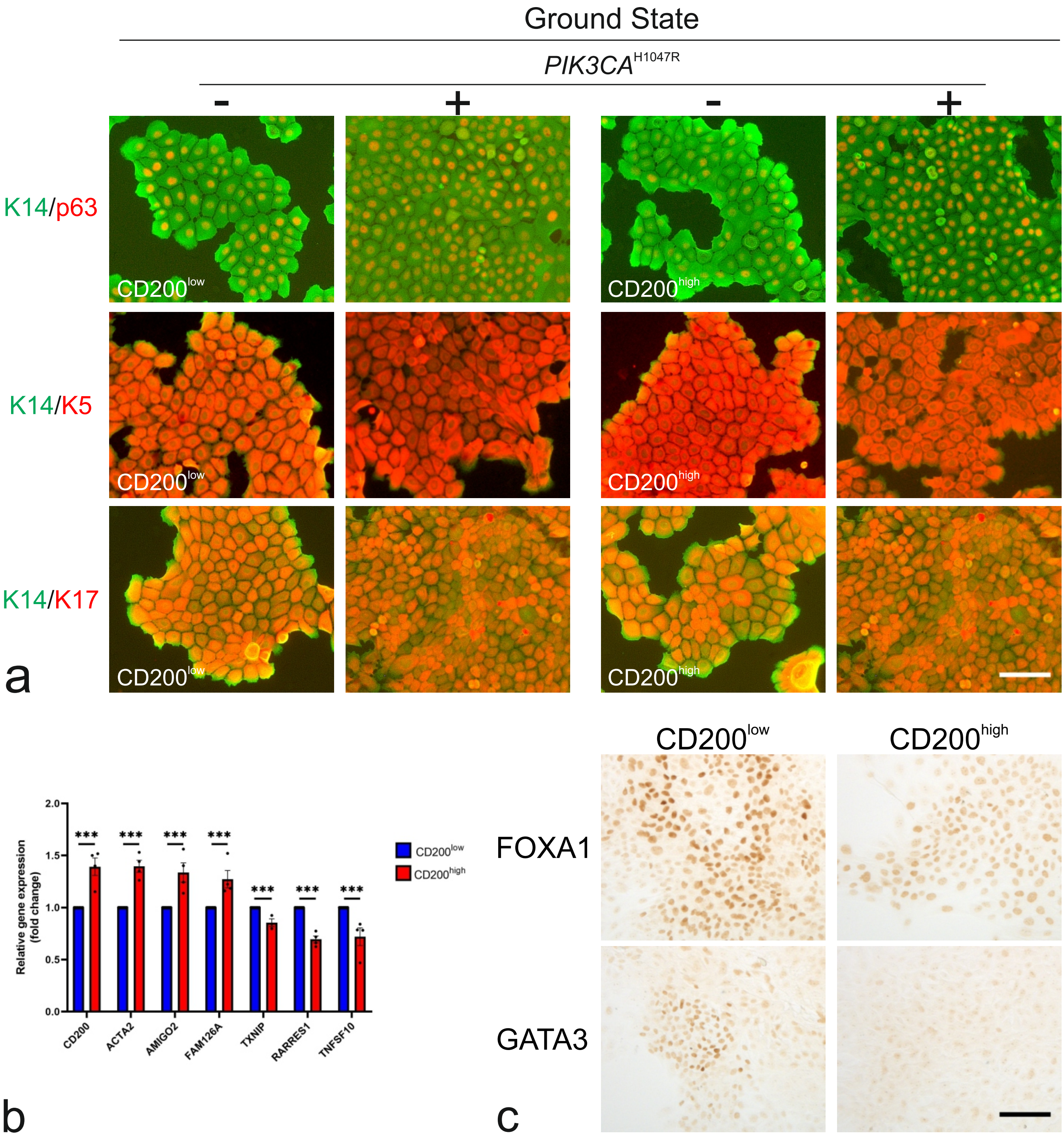

Supplementary Fig. 7. Mutant* PIK3CA *transformed CD200^{low} and CD200^{high} MEP cells maintain correct polarization.

Micrographs of fluorescence staining of cryosections stained for integrin β4 (ITGB4, green), mucin 1 (MUC1, red) and nuclei (blue) of structures formed in rBM by CD200^{low}-*hTERT*-shp53-*PIK3CA*^{H1047R} (CD200^{low}) and CD200^{high}-*hTERT*-shp53-*PIK3CA*^{H1047R} (CD200^{high}) cells (scale bar, 50 μm).

Supplementary Fig. 7. Mutant* PIK3CA *transformed CD200^{low} and CD200^{high} MEP cells maintain correct polarization

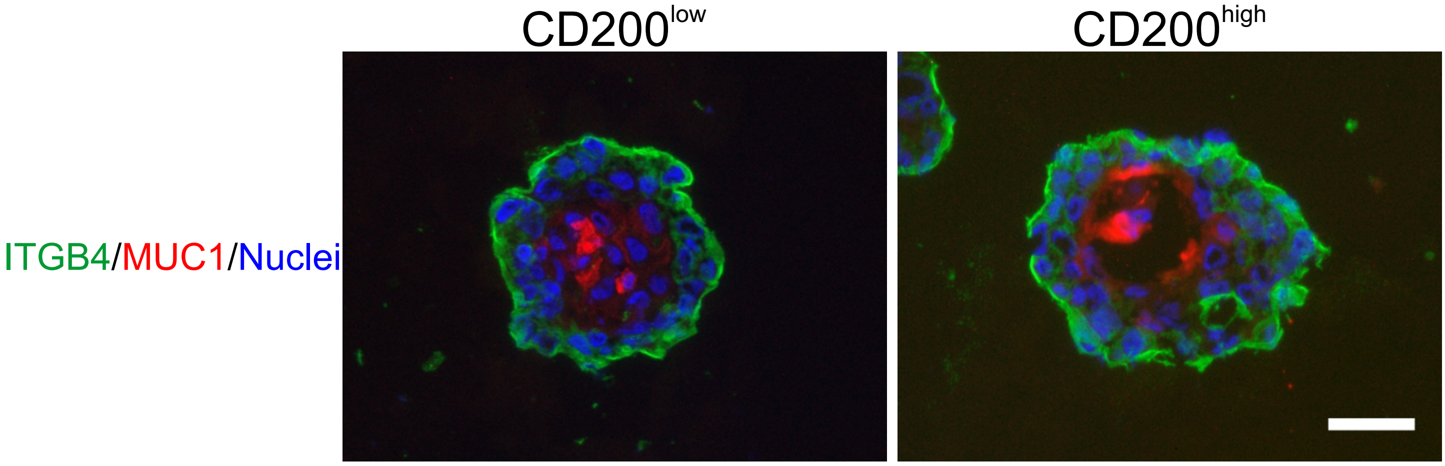

Supplementary Table 1. Overview of cell numbers and their anatomical origins in MEP clusters.

Table shows absolute cell numbers and percentages per cluster as well as cell numbers contributed from TDLUs and ducts.

Supplementary Table 1. Overview of cell numbers and their anatomical origins in MEP clusters

Cluster	Cell number	%	TDLU-derived	Duct-derived
0	3670	19.6%	2146	1524
1	3608	19.3%	2087	1521
2	3071	16.4%	1583	1488
3	2641	14.1%	1880	761
4	2534	13.6%	1744	790
5	2322	12.4%	1233	1089
6	449	2.4%	264	185
7	174	0.9%	50	124
8	158	0.8%	113	45
9	51	0.3%	33	18
**Sum**	**18678**	**100%**	**11133**	**7545**

Supplementary Table 2*. *Overview of estimated cell numbers from TDLUs and ducts from each of three biopsies.

Table shows donor age, estimated cell number before and after quality control (QC) and filtration as well as median genes per cell before and after QC and filtration for scRNA-seq.

Supplementary table 2. Overview of estimated cell numbers from TDLUs and ducts from each of three biopsies

Biopsy	Age	Location	Estimated number of cells	Number of cells after QC and filtration	Median genes per cell	Median genes per cell after QC and filtration
1	18	TDLUs	4,034	3,617	1,256	1,278
		Ducts	4,766	4,647	1,057	1,058
2	18	TDLUs	6,821	6,398	1,130	1,142
		Ducts	1,742	1,258	2,054	1,905
3	18	TDLUs	3,653	1,640	2,209	1,941
		Ducts	3,230	1,118	1,961	2,057
Total			24,246	18,678		1,259

Supplementary Table 3. Overview of antibodies used for immunofluorescence staining of cultured cells and tissue sections.

Table lists clones, companies, catalogue numbers, and dilutions of antibodies used for immunofluoresence.

Supplementary Table 3. Overview of antibodies used for immunofluorescence staining of cultured cells and tissue sections

Antigen	Clone	Company	Cat. No.	Dilution
K14	LL002	Monosan	MONX10687	1:100
α-SMA	1A4	Sigma-Aldrich	A-2547	1:500
α-/γ-SMA	HHF35	Enzo	ENZ-30931	1:25
K19	Ba16	Abcam	ab20210	1:100/1:300
K19	A53-B/A2	Abcam	ab7754	1:300
K17	E3	Dako	M7046	1:100
CD200	EPR22412229	Abcam	ab254193	1:50
AMIGO2	S86-36	Novus Biologicals	NBP2-22413	1:100
K5	XM26	Novocastra	NCL-CK5	1:250
K8/18	Troma-I	DSHB	Troma-I	1:100
P63	7JUL	Novocastra	NCL-L-P63	1:50
K7	OV-TL 12/30	Dako	M7018	1:300
ITGB4	3E1	Chemicon	MAB1964	1:500
MUC1	115D8	Biogenesis	0200-0101	1:10
ER	SP1	Epredia	RM-9101-S	1:25
FOXA1	JF10-02	Invitrogen	MA5-32556	1:200
GATA3	HG3-31	Santa Cruz	sc-268	1:200
AF488 Anti-mouse IgG1		Invitrogen	A21121	1:500
AF488 Anti-mouse IgG2b		Invitrogen	A21141	1:500
AF488 Anti-mouse IgG3		Invitrogen	A21151	1:500
AF568 Anti-mouse IgG1		Invitrogen	A21124	1:500
AF568 Anti-mouse IgG2a		Invitrogen	A21134	1:500
AF568 Anti-mouse IgG2b		Invitrogen	A21144	1:500
AF568 Anti-rat		Molecular Probes	A11077	1:500
AF633 Anti-mouse IgG3		Molecular Probes	A21156	1:500
