# Supplementary material for: Myoepithelial progenitors as founder cells of hyperplastic human breast lesions upon PIK3CA transformation
Source: Commun Biol. 2022 Mar 10;5:219. doi: 10.1038/s42003-022-03161-x (PMC8913783; doi:10.1038/s42003-022-03161-x)
Supplement: Supplementary file 3 — Description of Additional Supplementary Files [file 42003_2022_3161_MOESM3_ESM.pdf]

Description of Additional Supplementary Files

File name: Supplementary Data 1
Description: DEGs in clusters 0 to 9.

File name: Supplementary Data 2
Description: DEGs after exclusion of contaminating clusters 7, 8, and 9 including DEGs encoding surface proteins and DEGs specific for anatomic origin.

File name: Supplementary Data 3
Description: Source data for graphs and charts.
