# Supplementary material for: Myoepithelial progenitors as founder cells of hyperplastic human breast lesions upon PIK3CA transformation
Source: Commun Biol. 2022 Mar 10;5:219. doi: 10.1038/s42003-022-03161-x (PMC8913783; doi:10.1038/s42003-022-03161-x)
Supplement: Supplementary file 7 — Reporting Summary [file 42003_2022_3161_MOESM7_ESM.pdf]

nature portfolio

Corresponding author(s): Ole William Petersen

Last updated by author(s): Dec 9, 2021

Reporting Summary

Nature Portfolio wishes to improve the reproducibility of the work that we publish. This form provides structure for consistency and transparency in reporting. For further information on Nature Portfolio policies, see our Editorial Policies and the Editorial Policy Checklist.

Statistics

For all statistical analyses, confirm that the following items are present in the figure legend, table legend, main text, or Methods section.

n/a	Confirmed	
☐	☒	The exact sample size (*n*) for each experimental group/condition, given as a discrete number and unit of measurement
☐	☒	A statement on whether measurements were taken from distinct samples or whether the same sample was measured repeatedly
☐	☒	The statistical test(s) used AND whether they are one- or two-sided<br>*Only common tests should be described solely by name; describe more complex techniques in the Methods section.*
☒	☐	A description of all covariates tested
☐	☒	A description of any assumptions or corrections, such as tests of normality and adjustment for multiple comparisons
☐	☒	A full description of the statistical parameters including central tendency (e.g. means) or other basic estimates (e.g. regression coefficient) AND variation (e.g. standard deviation) or associated estimates of uncertainty (e.g. confidence intervals)
☐	☒	For null hypothesis testing, the test statistic (e.g. *F*, *t*, *r*) with confidence intervals, effect sizes, degrees of freedom and *P* value noted<br>*Give P values as exact values whenever suitable.*
☒	☐	For Bayesian analysis, information on the choice of priors and Markov chain Monte Carlo settings
☒	☐	For hierarchical and complex designs, identification of the appropriate level for tests and full reporting of outcomes
☒	☐	Estimates of effect sizes (e.g. Cohen's *d*, Pearson's *r*), indicating how they were calculated

Our web collection on statistics for biologists contains articles on many of the points above.

Software and code

Policy information about availability of computer code

Data collection	Gene set enrichment analysis was performed using the data deposited in the Molecular Signatures Database (https://www.gsea-msigdb.org/gsea/msigdb/).<br>The softwares Leica Application Suite (version 4.5.0) by Leica Microsystems and Zen (version 3.2) by Zeiss were used for image acquisition.<br>The software BD FACSDiva (version 8.0.1) By BD Biosciences was used to aquire FACS data.
Data analysis	The software FCS express 6 Flow Research (version 6.06.0033) by De Novo Software was used to analyze FACS data.<br>The software Cell Ranger (version 2.1.0) by 10x Genomics and the R package Seurat (version 3.0, R version 3.6.2) were used to analyze scRNA-seq data.<br>Gene set enrichment analysis was performed using the "Invesigate Gene Sets" function of the Molecular Signatures Database (https://www.gsea-msigdb.org/gsea/msigdb/).<br>Image analysis was performed using Fiji (version 1.51s).<br>The software Bio-Rad CFX Maestro 1.0 (version 4.0) by Bio-Rad was used to analyze qRT-PCR data.<br>Soft agar assays were analyzed using the Intellesis function of the software ZEN blue (version 3.2) by Zeiss.<br>Statistical analyses were performed using GraphPad Prism (version 9.0.0).

For manuscripts utilizing custom algorithms or software that are central to the research but not yet described in published literature, software must be made available to editors and reviewers. We strongly encourage code deposition in a community repository (e.g. GitHub). See the Nature Portfolio guidelines for submitting code & software for further information.

Data

Policy information about availability of data

All manuscripts must include a data availability statement. This statement should provide the following information, where applicable:

- Accession codes, unique identifiers, or web links for publicly available datasets
- A description of any restrictions on data availability
- For clinical datasets or third party data, please ensure that the statement adheres to our policy

All data are included in the article and supporting information. Raw data can be obtained from the authors upon reasonable request.

Field-specific reporting

Please select the one below that is the best fit for your research. If you are not sure, read the appropriate sections before making your selection.

☒ Life sciences ☐ Behavioural & social sciences ☐ Ecological, evolutionary & environmental sciences

For a reference copy of the document with all sections, see nature.com/documents/nr-reporting-summary-flat.pdf

Life sciences study design

All studies must disclose on these points even when the disclosure is negative.

Sample size	For all experiments n = 3 was chosen as minimal replicate number. For experiments involving primary human tissue that exhibited biopsy-dependent variability, higher sample sizes were chosen as indicated in the figure legends. For scRNA-seq, we reasoned that >15,000 cells were sufficient to detect rare cell populations, for example stem cells that are described to comprise approximately 4% (600 cells of 15,000) of myoepithelial cells in the human breast as estimated by limited dilution transplantation assays.
Data exclusions	Clusters 7, 8, and 9 were excluded from analysis of scRNA-seq data because they constituted contamination with stromal cells as shown in supplementary figure 1B and 1C. When determining the location of CD200 protein in sections of primary breast tissue, sections that did not contain epithelial cells were excluded from analysis.
Replication	All replication attempts were successful after adjusting media compositions for cell culture and antibody dilutions and fixation methods for immunohistochemistry, immunocytochemistry, and FACS. However, some degree of variability was observed in experiments involving primary human tissue as expected.
Randomization	Biopsies for immunohistochemistry and FACS were chosen randomly from the lab's repository. Biopsies for scRNA-seq were chosen based on donor age (three 18-year old, age-matched biopsies were randomly selected). For immortalization and transformation of myoepithelial cells, we selected a biopsy with high content of CD200low myoepithelial cells that expressed high levels of alpha smooth muscle actin.
Blinding	Blinding was not relevant for neither scRNA-seq data analysis since the data was analyzed using an unsupervised data processing pipeline nor for image analysis because automatic thresholds were used to identify positively-stained cells.

Reporting for specific materials, systems and methods

We require information from authors about some types of materials, experimental systems and methods used in many studies. Here, indicate whether each material, system or method listed is relevant to your study. If you are not sure if a list item applies to your research, read the appropriate section before selecting a response.

Materials & experimental systems

n/a	Involved in the study	
☐	☒	Antibodies
☐	☒	Eukaryotic cell lines
☒	☐	Palaeontology and archaeology
☐	☒	Animals and other organisms
☐	☒	Human research participants
☒	☐	Clinical data
☒	☐	Dual use research of concern

Methods

n/a	Involved in the study	
☒	☐	ChIP-seq
☐	☒	Flow cytometry
☒	☐	MRI-based neuroimaging

Antibodies

Antibodies used	anti-Trop2-bv421 and bv510 from BD Biosciences, clone 162-46, cat. no. 563243/563244, dilution 1:50<br>anti-CD271- PE from BioLegend, clone Me20.4, cat. no. 345106, dilution 1:50<br>anti-CD271-APC from Cedarlane Laboratories, clone Me20.4, cat. no. CL10013APC, dilution 1:50

	anti-CD200-APC or bv421 from BioLegends, clone Ox-104, cat. no. 329207/329209, dilution 1:50<br>anti-AMIGO2-AF488 from Novus Biologicals, clone S86-36, cat. no. NBP2-22413AF488, dilution 1:50<br>anti-K14 from Monosan, clone LL002, cat. no. MONX10687, dilution 1:100<br>anti-alpha smooth muscle actin from Sigma, clone 1A4, cat. no. A-2547, dilution 1:500<br>anti-smooth muscle actin from Enzo, clone HHF35, cat. no. ENZ-30931, dilution 1:25<br>anti-K19 from Abcam, clone Ba16, cat. no. ab20210, dilution 1:100/1:300<br>anti-K19 from Abcam, clone A53-B/A2, cat no. ab7754, dilution 1:300<br>anti-K17 from Dako, clone E3, cat. no. M7046, dilution 1:100<br>anti-CD200 from Abcam, clone EPR22412229, cat. no. ab254193, dilution 1:50<br>anti-AMIGO2 from Novus Biologicals, clone S86-36, cat. no. NBP2-22413, dilution 1:100<br>anti-K5 from Novocastra, clone XM26, cat. no. NCL-CK5, dilution 1:250<br>anti-K8/18 from DSHB, clone Troma-I, cat. no. Troma-I, dilution 1:100<br>anti-p63 from Novocastra, clone 7Jul, cat. no. NCL-L-P63, dilution 1:50<br>anti-K7 from Dako, clone OV-TL 12/30, cat. no. M7018, dilution 1:300<br>anti-Integrin beta 4 from Chemicon, clone 3E1, cat. no. MAB1967, dilution 1:500<br>anti-Muc1 from Biogenesis, clone 115D8, cat. no. 0200-0101, dilution 1:10<br>AF488 anti mouse IgG1 from Invitrogen, cat. no. 21121, dilution 1:500<br>AF488 anti-mouse IgG2b from Invitrogen, cat. no. A21141, dilution 1:500<br>AF488 anti-mouse IgG3 from Invitrogen, cat. no. A21151, dilution 1:500<br>AF568 anti-mouse IgG1 from Invitrogen, cat. no. A21124, dilution 1:500<br>AF568 anti-mouse IgG2a from Invitrogen, cat. no. A21134, dilution 1:500<br>AF568 anti-mouse IgG2b from Invitrogen, cat. no. A21144, dilution 1:500<br>AF568 anti-rat from Molecular Probes, cat. no. A11077, dilution 1:500<br>AF633 anti-mouse IgG3 from Molecular Probes, cat. no. A21156, dilution 1:500
Validation	For FACS, controls where antibodies were omitted were included in all experiments. Furthermore, FMO controls were performed for all antibodies.<br>For immunofluorescence, each antibody was validated by performing control stainings with secondary antibody only, doing dilution series, and doing control stainings with same isotype antbodies.

Eukaryotic cell lines

Policy information about cell lines

Cell line source(s)	Primary human breast myoepithelial cells immortalized with hTERT, shp53, and/or PIK3CA(H1047R)
Authentication	None of the cell lines used were authenticated.
Mycoplasma contamination	All cell lines tested negative for Mycoplasma contamination.
Commonly misidentified lines<br>(See ICLAC register)	N/A

Animals and other organisms

Policy information about studies involving animals; ARRIVE guidelines recommended for reporting animal research

Laboratory animals	6-10 week old female NOD.Cg-Prkdc(SCID)Il2rg(tm1sug) (NOG) mice (Taconic)
Wild animals	The study did not include any wild animals.
Field-collected samples	The study did not contain any samples collected from the field.
Ethics oversight	All experiments involving animals have been approved by the Danish Animal Experiments Inspectorate with reference to 2017-15-0201-01315.

Note that full information on the approval of the study protocol must also be provided in the manuscript.

Human research participants

Policy information about studies involving human research participants

Population characteristics	Breast tissue was collected from healthy females undergoing reduction mammoplasties for cosmetic reasons. Except for the age of the donors, no information was available about the patients.
Recruitment	Recruitment was performed by the private hospital Capio CFR Hellerup (Hellerup, Denmark).
Ethics oversight	The Regional Scientific Ethical Committees (Region Hovedstaden, H-2-2011-052) and the Danish Data Protection Agency (2011-41-6722) reviewed and approved the use and storage of human material.

Note that full information on the approval of the study protocol must also be provided in the manuscript.

Flow Cytometry

Plots

Confirm that:

☒ The axis labels state the marker and fluorochrome used (e.g. CD4-FITC).

☒ The axis scales are clearly visible. Include numbers along axes only for bottom left plot of group (a 'group' is an analysis of identical markers).

☒ All plots are contour plots with outliers or pseudocolor plots.

☒ A numerical value for number of cells or percentage (with statistics) is provided.

Methodology

Sample preparation	Frozen breast organoids from primary reduction mammoplasties were thawed by placing the cryogenic vial with the organoids in warm ethanol for 1-2 min at 37 ºC and 60 rpm. Organoids were carefully transferred to a fresh 15 ml conical tube with 10 ml DMEM/F12 and spun down for 1 min at 1,600 rpm. The pellet was resuspended in 3-4 ml 0.25% trypsin in PBS with 100 mM EDTA. Organoids were trypsinized for 6-8 min at 37 ºC and 160 rpm and trypsinization was stopped by the addition of 0.5-1 ml FCS. The resulting cell solution was sheared through a 100 µm filter and spun down for 3 min at 1,500 rpm. The pellet was resuspended in cold HEPES buffer and antibodies or nothing (control) were added. Cells were incubated with antibodies for 45 min at 4 ºC. Subsequently, tubes were filled with cold HEPES buffer and spun down for 3 min at 1,500 rpm. The pellet was resuspended in 0.4-2 ml HEPES buffer depending on the pellet size and filtered through a 20 µm cup filter to obtain a single cell solution. Fixable Viability Stain 780 (BD Horizon, 565388) was added according to the manufacturer's instructions to mark dead cells. Cells were then subjected to analysis and/or sorting. Prior to sorting multicolor compensations were set up.
Instrument	FACSAria™ Fusion Cytometer and FACSAria™ IIu Cytometer (both BD)
Software	Data was aquired using the software BD FACSDiva (version 8.0.1) By BD Biosciences, while FCS Express 6 Flow Research (version 6.06.0033) by De Novo software was used for data analysis.
Cell population abundance	Myoepithelial cells comprised 10-40% of living cells in all samples depending on the biopsy. CD200low cells accounted for 5-15% of myoepithelial cells depending on the biopsy (see Supplementary Figure 2A), while 10-15% of myoepithelial cells expressing the highest levels of CD200 were sorted as CD200high cells.
Gating strategy	FSC-A/SSC-A was used to exclude debris, while SSC-H/SSC-W and FSC-H/FSC-W gates were used to remove duplets. FCS-A/Fixable Viability Stain (780) was used to deplete dead cells (Fixable Viability stain low). Myoepithelial cells were recognized as Trop2high/CD271high. CD200low and CD200high myoepithelial cells were isolated as described above.

☐ Tick this box to confirm that a figure exemplifying the gating strategy is provided in the Supplementary Information.
